# Uric acid regulates α-synuclein transmission in Parkinsonian models

**DOI:** 10.3389/fnagi.2023.1117491

**Published:** 2023-08-28

**Authors:** Yu Jin Shin, Yeon Ju Kim, Ji Eun Lee, Yi Seul Kim, Jung Wook Lee, HyeonJeong Kim, Jin Young Shin, Phil Hyu Lee

**Affiliations:** ^1^Department of Neurology, Yonsei University College of Medicine, Seoul, Republic of Korea; ^2^Severance Biomedical Science Institute, Yonsei University, Seoul, Republic of Korea; ^3^Department of Medical Science, Catholic Kwandong University College of Medicine, Gangneung-si, Republic of Korea

**Keywords:** uric acid, α-Syn, transmission, endocytosis, Parkinson’s disease

## Abstract

Ample evidence demonstrates that α-synuclein (α-syn) has a critical role in the pathogenesis of Parkinson’s disease (PD) with evidence indicating that its propagation from one area of the brain to others may be the primary mechanism for disease progression. Uric acid (UA), a natural antioxidant, has been proposed as a potential disease modifying candidate in PD. In the present study, we investigated whether UA treatment modulates cell-to-cell transmission of extracellular α-syn and protects dopaminergic neurons in the α-syn-enriched model. In a cellular model, UA treatment decreased internalized cytosolic α-syn levels and neuron-to-neuron transmission of α-syn in donor-acceptor cell models by modulating dynamin-mediated and clathrin-mediated endocytosis. Moreover, UA elevation in α-syn-inoculated mice inhibited propagation of extracellular α-syn which decreased expression of phosphorylated α-syn in the dopaminergic neurons of the substantia nigra leading to their increased survival. UA treatment did not lead to change in markers related with autophagolysosomal and microglial activity under the same experimental conditions. These findings suggest UA may control the pathological conditions of PD via additive mechanisms which modulate the propagation of α-syn.

## Introduction

Parkinson’s disease (PD) is a common neurodegenerative disorder pathologically characterized by loss of dopaminergic neurons in the substantia nigra pars compacta (SNpc) and the presence of Lewy bodies and Lewy neurites, which mainly consist of aggregates of α-synuclein (α-syn). Intracellular proteinaceous aggregates of α-syn, which can be monomeric, oligomeric intermediate, or fibrillar, have a critical role in the pathogenesis of PD (Eschbach and Danzer, 2014). Recent studies suggest that α-syn can propagate from one area of the brain to others via cell-to-cell transmission, which might be the underlying mechanism for pathological propagation of Lewy bodies and disease progression of motor and non-motor symptoms (Lee et al., 2005; Desplats et al., 2009; Hansen et al., 2011; Gaugler et al., 2012). α-Syn may be transmitted via endocytosis to neighboring neurons, which is known to play a key role in spreading of α-syn (Lee et al., 2008; Cheng et al., 2011; Oh et al., 2016). Several studies also provided evidence of cell surface receptor-mediated endocytosis of α-syn such as FcγRIIB or lymphocyte activation gene-3 (Mao et al., 2016; Choi et al., 2018). Moreover, we have previously demonstrated that α-syn may be internalized and propagated via interaction with *N*-methyl-D-aspartate receptors (Lee et al., 2021). Thus, the modulation of α-syn propagation would be an important strategy to delay the progression of PD as one of disease modifying therapeutics.

Uric acid (UA) is an important natural antioxidant in brain tissue and blood that is produced as a byproduct of purine metabolism. Preclinical studies have demonstrated that the neuroprotective effects of UA by scavenging oxygen radicals and reactive nitrogen and chelating metal ions in animal models (Davies et al., 1986; Yu et al., 1998; Schlesinger and Schlesinger, 2008; Huang et al., 2017; Bi et al., 2018). Clinical studies have demonstrated that serum UA levels are much lower in patients with PD compared to those without. Moreover, several epidemiological studies have demonstrated that higher UA levels are linked to reduced risk of PD and slow PD progression after the diagnosis of PD (Davis et al., 1996; de Lau et al., 2005; Weisskopf et al., 2007; Schwarzschild et al., 2008; Ascherio et al., 2009; Gao et al., 2016). These findings suggest that UA may have neuroprotective properties against PD-associated microenvironment, possibly acting as a disease modifier in PD. In the present study, we hypothesized that UA may exert neuroprotective effects via regulation of α-syn transmission in Parkinsonian models. To prove this, we triggered α-syn pathology by intrastriatal injection of preformed α-syn fibrils (PFF) in mice, where the injection of the α-syn has been shown to spread to other anatomically interconnected brain regions, including the dorsal motor nucleus of the vegal nerve, locus coeruleus, and substantia nigra pars compacta. Previous animal studies have reported progressive loss of dopaminergic neurons in substantia nigra and behavioral deficits between 30 and 180 days after intrastriatal injection of PFFs (Luk et al., 2012; Tarutani et al., 2018). We investigated whether treatment of UA modulates the cell-to-cell transmission of extracellular α-syn and protects dopaminergic neurons in α-syn enriched cellular and animal PD models.

## Materials and methods

### SH-SY5Y culture

The human neuroblastoma cell line SH-SY5Y was obtained from the Korean Cell Line Bank (Seoul National University, Seoul, Republic of Korea). SH-SY5Y cells were maintained in Dulbecco’s Modified Eagle Medium (DMEM, HyClone) supplemented with 10% fetal bovine serum (JC Bio) and a mixture of penicillin and streptomycin (1%, HyClone). When the cells reached 70–80% confluence, they were trypsinized and subcultured, and maintained at a temperature of 37°C in a humidified atmosphere containing 5% CO2/air. For differentiation, SH-SY5Y cells were plated at a density of. The next day, the cells were incubated in fresh DMEM with 10 μM of retinoic acid (Sigma). The medium was replaced on alternate days and the cells were allowed to differentiate for 5 days. The differentiated cells were then pretreated with UA (Sigma, 400 μM) for 24 h prior to treatment with α-syn (1 μM). Lysosomal inhibitor, bafilomycin A1 (Sigma, 50 nM) was added to the SH-SY5Y cells to exclude the function of lysosomal degradation.

### Preformed fibrillary α-syn preparation

Recombinant human α-syn [5 mg/ml in phosphate buffered saline (PBS) containing 50 mM Tris and 100 mM NaCl] was agitated at 37°C (1000 rpm) for 5 days. After then, fragmentation of α-syn fibrils was carried out by sonication using a Qsonica 4423 Q55 sonicator with 5/64-inch probe tip and sonicated at 20% amplitude, for a total time of 2 min (10 s pulse on/off). The α-syn fibrils were then visualized using electron microscopy. The confirmed preformed fibrillar protein was briefly sonicated to allow it to readily internalize into cells. The α-syn fibrils were visualized after agitation using electron microscopy.

### Cell viability analysis

SH-SY5Y cells were plated in 24-well polystyrene plates (SPL Life Sciences) at a density of and incubated at 37°C for 24 h to allow cells to stabilize. SH-SY5Y cells were then treated with various concentration of UA. Simultaneously, the same volume of fresh DMEM was added to the control groups. Plates were incubated at 37°C for a 24. Cell viability was measured using a cell proliferation assay (CellTiter 96^®^ AQueous One Solution Cell Proliferation Assay, [Promega]) in accordance with the manufacturer’s protocol. Mixture of a tetrazolium compound [3-(4,5-dimethylthiazol-2-yl)-5-(3-carboxymethoxyphenyl)-2-(4-sulfophenyl)-2H-tetrazolium, inner salt; MTS] and an electron coupling reagent (phenazine ethosulfate; PES) was then added to a final concentration of 0.5 mg/ml. After incubation for 1 h, the medium was transferred to 96-well plates (SPL Life Sciences) in triplication and absorbance was measured by using the ELISA microplate reader (VersaMax, Molecular Devices, USA) at 490 nm.

### Bimolecular fluorescence complementation (BiFC) system

SH-SY5Y cells were transfected with Venus1-αSyn (V1S) or αSyn–Venus2 (SV2) plasmid using electroporation. Stable cell lines were maintained with 200 μg/ml G418 (Invitrogen) (Bae et al., 2014). These two stable cell lines express αSyn fused to either the amino (N) terminus (V1S) or carboxy (C) terminus (SV2) fragment of Venus, a variant of yellow fluorescence protein. When the two cell lines were co-cultured, fluorescence resulting from dimerization or oligomerization of the V1S and SV2 fusion proteins (Outeiro et al., 2008; Goncalves et al., 2010) during cell-to-cell transfer of αSyn was visualized using BiFC system. V1S and SV2 cell lines were plated in separate 60-mm dish (SPL Life Sciences) at a density of 6 cm × 10^6/^cm and incubated at 37°C for 24 h. Simultaneously, UA-treated co-culture group was incubated with UA (400 μM) during 24 h stabilization. After stabilization, V1S and SV2 cell lines were subcultured for co-culture. V1S and SV2 stable cells were mixed in a coverslip at a density of 6 cm × 10^6/^cm and cultured for 48 h before visualization.

### Immunocytochemistry

SH-SY5Y cells were washed three times using PBS and incubated 0.1% Triton X-100 for 10 min. They were fixed with 4% paraformaldehyde for 20 min at room temperature. After then, cells were washed three times and they were blocked with 0.5% bovine serum albumin for 1 h at room temperature. After blocking, they were incubated overnight at 4°C with specific primary antibodies. The primary antibodies that were used for staining are as follows: rabbit anti-α-syn (Abcam, ab138501), mouse anti-clathrin (Abcam, ab2731), rabbit anti-Rab5 (Abcam, ab18211). Immunofluorescence labeling was carried out by incubating cells for 2 h in donkey anti-mouse IgG (Alexa 488, green) and goat anti-rabbit IgG (Alexa 647, red) secondary antibodies. Cell nuclei were counterstained with 4′, 6-diamidino-2-phenylindole (DAPI; Invitrogen, D1306). The immunostained cells were visualized using a Zeiss LSM 700 confocal imaging system. Quantification of the fluorescence intensities of α-syn, clathrin, and Rab5 was performed through Zen software program (Zeiss).

### Animal study

All procedures were performed in accordance with the Laboratory Animals Welfare Act, the Guide for the Care and Use of Laboratory Animals, and the Guidelines and Policies for Rodent Experiments provided by the Institutional Animal Care and Use Committee (IACUC) at the Yonsei University Health System. Male C57BL/6 mice (Orient Bio) were acclimated in a climate-controlled room with a constant 12 h light/dark cycle for 1 week prior to drug administration. The mice were divided into the following three groups (*n* = 10 per group): (1) control group; (2) only α-syn-treated group; and (3) UA-elevated group in α-syn-treated mice. To elevate UA serum levels, the mice were administered a daily intraperitoneal (IP) injection of potassium oxonate (KOx; 500 mg/kg; Sigma) and guanine monophosphate (GMP; 500 mg/kg; Sigma) for 2 weeks, whereas the control group mice received normal saline. To construct Parkinsonian model, Preformed fibrillary α-syn (1 μl, total 5 μg/hemisphere) was slowly injected into the striatum bilaterally (0.2 mm posterior to the bregma, ± 2.0 mm lateral to the midline, and −2.6 mm ventral to the brain surface) using a stereotaxic apparatus. After α-syn inoculation, the UA-elevated group received a daily IP injection for additional 4 weeks (total 6 weeks). All mice were sacrificed 1 month after α-syn inoculation (Figure 3A).

### Preparation of serum and brain tissue

All mice were deeply anesthetized by isoflurane inhalation at the end of the experimental period and their blood and brains for western blotting analysis were collected. To measure serum UA levels, blood samples from the abdominal aorta were collected in serum separator tube (BD Diagnostic Systems, Sparks, MD, USA). Serum and blood cells were separated by centrifugation (16,000 rcf, 20 min), then immediately frozen and stored at −20°C until analysis. For immunohistochemistry, mice were perfused with 4% paraformaldehyde. Brains were carefully harvested from the skulls, post-fixed overnight in 4% paraformaldehyde, and stored in 30% sucrose in PBS for 1–2 days at 4°C until they sank. Coronal brain tissue sections (25 μm thickness) were cut using a cryostat and stored in sterile tissue storage solution (30% glycerol, 30% ethylene glycol, 30% distilled water, 10% 0.2 M PB) at 4°C until analysis.

### Rotarod test

To assess motor function and coordination, along with balance, mice were tested on the rotarod apparatus (MED-Associates, USA). On the day prior to initiating the training session, mice were habituated to the apparatus for 15 min. In training trials, mice were trained to run on the rotarod (20 rpm) for 10 min without falling, twice a day for three consecutive days prior to α-synuclein administration. In test trials, mice were placed on the rotarod at 30 rpm (cut-off time of 700 s maximum). The latency time to fall was recorded.

### Measurements of serum UA level

Each serum samples obtained from mice were used for measuring serum UA level. Enzyme colorimetric assay was performed by Seoul Clinical Laboratories (SCL). Uricase converts UA into allantoin and hydrogen peroxide. In the presence of hydrogen peroxide, 4-aminophenazone is oxidized by hydrogen peroxide and become quinone-diimine. The color intensity of generated quinone-diimine is directly proportional to the concentration of UA. Absorbance was measured at a 552 nm wavelength using a Cobas 8000 c502 (Roche Diagnostics [HITACHI], Japan).

### Western blotting analysis

Cells harvested from the cell culture plates for western blotting analysis were dissolved in ice-cold Radio-Immunoprecipitation Assay (RIPA) buffer (50 mM Tris–HCl, pH 7.5, with 150 mM sodium chloride, 1% triton X-100, 1% sodium deoxycholate, 0.1% SDS, and 2 mM EDTA sterile solution; Lugen Sci Co.) plus protease inhibitors and phosphatase inhibitors (Xpert Duo inhibitor cocktail solution; GenDEPOT). Mice were anesthetized with isoflurane and their brains were carefully collected for western blotting analysis as mentioned in “Preparation of serum and brain tissue” section. Brain tissue was homogenized and also dissolved in RIPA buffer for protein extraction. Lysates were centrifuged (13,000 rpm at 4°C) for 20 min (13,000 rpm), and supernatants were transferred to sterile tubes. Briefly, 10 or 20 μg of protein were separated by SDS-gel electrophoresis and transferred to hydrophobic polyvinylidene difluoride (PVDF) membranes (GE Healthcare) which were blocked in 5% skim milk in PBST (0.1% Tween 20). Membranes were probed with the following primary antibodies: mouse anti-actin (Santa Cruz, sc 47778), mouse anti-α-tubulin (Santa Cruz, sc-32293), rabbit anti-α-syn (Abcam, ab138501), rabbit anti-phospho S129 α-syn (Abcam, ab59264), anti-aggregated α-synuclein antibody (Millipore, MABN389), rabbit anti-dynamin (Santa Cruz, sc-11362), mouse anti-clathrin (Abcam, ab2731), rabbit anti-Rab5 (Abcam, ab18211), rabbit anti-EEA1 (Abcam, ab2900), rabbit anti-Iba-1 (Abcam, ab178847), mouse anti-SQSTM1/p62 (Abcam, ab56416), rabbit anti-LC3B (Sigma, L7543), rabbit anti-LAMP2 (BioVision, 3900-100). After overnight incubation with primary antibodies at 4°C, membranes were incubated with a secondary antibody conjugated with horse radish peroxidase for 2 h at room temperature. Antigen-antibody complexes were visualized with ECL solution (GenDEPOT), For quantitative analysis, immunoblotting band densities were measured by Image J.

### Immunohistochemistry

Brain sections were washed twice in 0.01% Triton X-100 (Sigma) and incubated in 0.5% Triton X-100 for 15 min at room temperature for permeabilization. Sections were blocked with 0.5% bovine serum albumin (BSA, Sigma) for 1 h, then washed twice in 0.01% Triton X-100 and incubated overnight 4°C with primary antibodies. The primary antibodies were: rabbit anti-α-syn (Abcam, ab138501), mouse anti-NeuN (Abcam, ab104224), rabbit anti-phosphorylated α-syn (Abcam, ab59264), and mouse anti-Tyrosine hydroxylase (TH) (Sigma, T2928). Immunofluorescence labeling was carried out by incubating tissue slides for 2 h in donkey anti-mouse IgG and goat anti-rabbit IgG (both Alexa Fluor-488, green and Alexa Fluor-647, red) secondary antibodies (1:200, Invitrogen). Cell nuclei were counterstained with 4′, 6-diamidino-2-phenylindole (DAPI; Invitrogen, D1306). For the TH staining of midbrain, the brain tissue was incubated in biotinylated secondary antibodies in blocking solution (1:400) for 2 h at room temperature. The TH antibodies were visualized using 0.05% diaminobenzidine (DAB, Dako, Carpinteria, CA, USA). The immunostained tissue samples were visualized using bright-field microscopy and immunofluorescence images were viewed with a Zeiss LSM 700 confocal imaging system (Jena, Germany). To analyze the localization of antigens in double stained tissues, immunofluorescence images were created from the same tissue sections and merged using Zeiss ZEN software. The fluorescence intensity was quantified using ZEN software program.

### Measurement of α-syn and phosphorylated α-syn

The mass of α-syn was measured using the sandwich ELISA kit (AnaSpec, USA) and the mass of phosphorylated α-syn was measured using the sandwich ELISA kit (MybioSource, USA). Cell culture medium collected from each experimental group (control, α-syn, and α-syn/UA) was used for ELISA analysis. Brain tissue was homogenized and also dissolved in RIPA buffer for protein extraction. Brain lysates were centrifuged (13,000 rpm at 4°C) for 20 min (13,000 rpm), and supernatants were transferred to sterile tubes. The supernatants were used for ELISA analysis. Each diluted sample and standard included in the kit were applied to microtiter strip plates precoated with antibody that specifically binds to α-syn or phosphorylated α-syn in cell culture medium and brain lysates. Diluted detection antibodies that were indirectly linked to an enzyme were applied to each sample diluent and standard. Following overnight incubation at 4°C, washing solution was added to each well and then aspirated six times. To ensure accurate optical reading, the plates were inverted and then patted until no moisture remained. 3,3′,5,5′-Tetramethylbenzidine (TMB) substrate solution was added to each well and incubated for 10 min until the blue gradient was clearly observed across the wells. Stop solution was added to each well until the color completely changed from blue to yellow. An automatic ELISA microplate reader (BioTek) was used with the wavelength setting at 450 nm. The Bio-Rad software was used to generate standard curves and to calculate the target antigen concentration of the samples.

### TH-positive cell counts

Tyrosine hydroxylase -immunostained neurons in the left and right SNpc of every fourth section were counted throughout the entire extent of the SNpc. Each stained tissue sample was visualized at low power and the number of TH-immunostained cells was counted at high power. To accurately count the number of TH-positive cells, TH-positive cells were counted only when the nucleus was optimally visualized, which occurred in only one focal plane. The average cell number of five mice in each experimental group was shown in graph.

### Statistical analysis

Mean differences among experimental groups were determined by one-way analysis of variance (ANOVA) followed by Bonferroni’s *post-hoc* test. Differences were considered statistically significant at *P* < 0.05. Statistical analysis was performed using the commercially available software SPSS Statistics 26 (IBM Corp: IBM SPSS Statistics for Windows, Armonk, NY, USA).

## Results

### UA modulates cell-to-cell transmission of extracellular α-syn in SH-SY5Y cells

**FIGURE 1:**
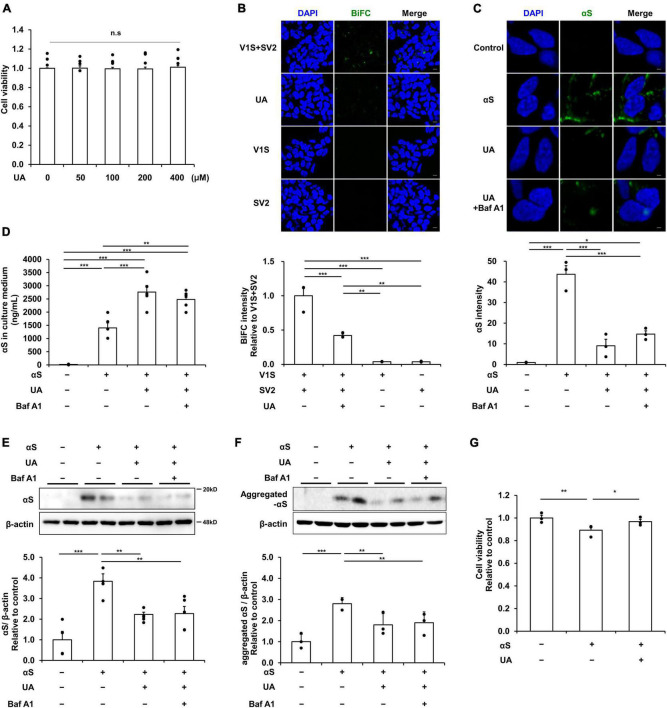
Uric acid (UA) modulates cell-to-cell transmission of extracellular α-syn in neuronal cells. **(A)** MTS analysis in UA-treated differentiated SH-SY5Y cells with a concentration of 0, 50, 100, 200, and 400 μM. UA treatment did not induce cytotoxicity within the set concentration range (*n* = 4 per group). **(B)** A donor-acceptor co-culture method for transmission of αS showing that the intensity of BiFC signals (green) were markedly decreased in the UA treatment group compared to the V1S and SV2 co-culture groups. Scale bar, 50 μm. **(C)** Immunostaining for internalization of αS fibrils (αS, green) and quantification of αS fluorescence intensity in SH-SY5Y cells among control, αS, and αS/UA-treated cells. Scale bar, 2 μm. **(D)** Quantification using ELISA analysis of extracellular αS in the culture medium of the control, αS, αS/UA, and αS/UA/bafilomycin-treated group (*n* = 5 per group). Western blot for αS **(E)** and aggregated αS **(F)** with control (*n* = 4), αS (*n* = 5), αS/UA (*n* = 5), and αS/UA/bafilomycin-treated (*n* = 5) groups. **(G)** MTS analysis in the control, αS, and αS/UA-treated group after incubation of αS fibrils for 24 h (*n* = 3 per group). Differences among conditions were evaluated by ANOVA with Bonferroni’s correction for multiple comparisons. All data are presented as mean ± SE. *P < 0.05, **P < 0.01, and ***P < 0.001.

Based on the cell viability test that UA treatment at various concentrations (0–400 μM) for 24 h did not affect the cell viability (Figure 1A), UA concentration was fixed as 400 μM. We utilized a BiFC system to directly demonstrate the effect of UA on transmission of α-syn between neuronal cells. BiFC intensity was markedly decreased in the UA treatment group compared to the V1S and SV2 co-culture groups. However, neither V1S-expressing cells nor SV2-expressing cells were fluorescent in the individual cultures (Figure 1B). To examine the modulatory effect of UA on internalization of α-syn fibrils, we treated UA to differentiated SH-SY5Y cells prior to α-syn incubation. Immunocytochemistry showed that treatment with UA significantly attenuated the immunoreactivity of α-syn compared to treatment with α-syn alone (Figure 1C). Western blotting also demonstrated that UA treatment decreased the expression of α-syn compared to α-syn alone (Figure 1E). To measure the amount of extracellular α-syn not internalized into cells, we performed ELISA analysis in culture medium harvested from each experimental group. The amount of extracellular α-syn in the UA treatment group was markedly higher than the α-syn alone group, presumably resulting from the inhibition of α-syn internalization (Figure 1D). Bafilomycin A1 was added to exclude lysosomal degradation of α-syn in neurons and resulted in no influence on α-syn internalization or extracellular α-syn levels. In addition, we characterized conformation-specific antibodies that preferentially recognize aggregated forms of α-syn fibril. As a results, the internalization of aggregated form of α-syn was also reduced by UA and bafilomycin A1 did not affect the levels of α-syn (Figure 1F and Supplementary Figure 1). The incubation of α-syn fibrils for 1 h decreased SH-SY5Y cell viability, but treatment of UA decreased α-syn-induced cell death compared to the α-syn alone (Figure 1G).

### UA inhibits cell-to-cell transmission of α-syn via blocking endocytic pathway

**FIGURE 2:**
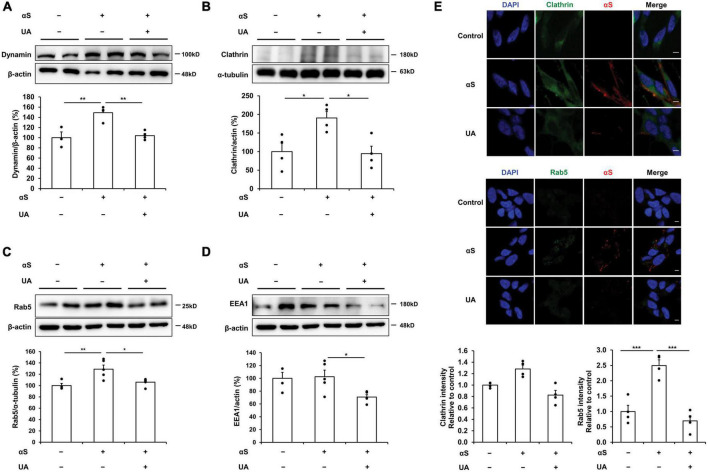
Uric acid (UA) inhibits cell-to-cell transmission of α-syn via blocking endocytic pathway. **(A)** Western blot for dynamin in control (*n* = 4), αS (*n* = 5), and αS/UA-treated (*n* = 5), neuronal cells and quantification graph. **(B)** Western blot for clathrin and quantification graph (*n* = 4 per group). **(C)** Western blot for Rab5, early endosome marker, and quantification graph (*n* = 4 for control, *n* = 5 for αS, and αS/UA-treated group). **(D)** Western blot for EEA1, a Rab5 effector protein, and quantification graph (*n* = 4 for control, *n* = 5 for αS-treated, and αS/UA-treated group). **(E)** Immunostaining of clathrin or Rab5 and αS with quantification of clathrin or Rab5 fluorescence intensity. Scale bar, 5 μm. Differences among conditions were evaluated by ANOVA with Bonferroni’s correction for multiple comparisons. All data are presented as mean ± SE. *P < 0.05, **P < 0.01, and ***P < 0.001.

We evaluated the expression of endocytic pathway markers to verify how UA blocks the internalization of α-syn fibrils in differentiated neuronal cells. Western blotting showed that the expression of dynamin was significantly increased in α-syn treated neuronal cells relative to control cells, whereas treatment with UA significantly decreased dynamin expression compared to cells treated with α-syn alone (Figure 2A). Similarly, α-syn-treated neuronal cells exhibited increased clathrin expression compared to control cells, whereas treatment with UA in α-syn-treated neuronal cells significantly attenuated the expression of clathrin. This indicates that UA suppresses clathrin-mediated endocytosis (CME) of α-syn (Figure 2B). The suppression of dynamin-mediated and clathrin-mediated endocytosis caused the expression of Rab5 localized with early endosomes to markedly decrease in UA-treated cells compared to cells treated with α-syn alone (Figure 2C). In addition, the expression of early endosome antigen, EEA1 (also known as “Rab5 effector”) which is involved in endosomal trafficking, decreased in UA-treated neuronal cells compared to α-syn alone-treated cells (Figure 2D). Moreover, immunocytochemistry showed that α-syn-treated neuronal cells increased the immunoreactivity of clathrin or Rab5 co-localized with α-syn, compared to control cells, whereas treatment with UA markedly decreased the immunoreactivity of clathrin or Rab5 compared to cells treated with α-syn alone (Figure 2E).

### UA elevation modulates α-syn transmission in the striatum of α-syn-inoculated mice

**FIGURE 3:**
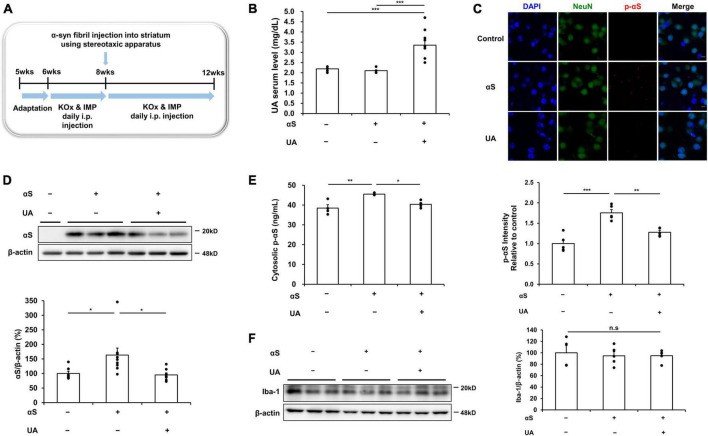
Uric acid (UA) elevation modulates α-syn transmission in the striatum of α-syn-inoculated mice. **(A)** Animal experiment schedule. **(B)** Serum UA levels were significantly higher in mice that were co-injected with GMP and KOx compared to control and αS-inoculated mice. **(C)** Immunohistochemical analysis showed that the immunoreactivity of p-αS in the striatal region was markedly increased in αS-inoculated mice, whereas the immunoreactivity of p-αS was significantly decreased in UA-elevated mice. Scale bar, 5 μm. **(D)** Western blotting showing that UA-elevated mice significantly attenuated the expression of cytosolic αS in the striatal region (*n* = 4 for control, *n* = 9 for αS-inoculated, and *n* = 8 for UA-elevated mice). **(E)** ELISA analysis showing that UA-elevated mice markedly decreased the amount of cytosolic p-αS in the striatal region compared to control and only αS-inoculated mice (*n* = 4 per group). **(F)** Western blotting showed that the expression of Iba-1 in the striatal region was comparable among control (*n* = 4), αS-inoculated (*n* = 5), and UA-elevated mice (*n* = 5). Differences among conditions were evaluated by ANOVA with Bonferroni’s correction for multiple comparisons. All data are presented as mean ± SE. *P < 0.05, **P < 0.01, and ***P < 0.001.

To examine the effect of UA elevation on α-syn transmission in α-syn-inoculated mice, mice were intraperitoneally co-injected with guanine 5′-monophosphate (GMP) and uricase inhibitor, potassium oxonate (KOx) to elevate their UA serum levels. Recombinant human α-syn fibrils were slowly inoculated bilaterally into the striatum. The detailed *in vivo* study design is illustrated in Figure 3A. Two weeks’ treatment with GMP and KOx led to a significant increase in the serum levels of UA (3.4 mg/dl) relative to the control mice (2.2 mg/dl) or only α-syn-inoculated mice (2.1 mg/dl) (Figure 3B). We then evaluated whether UA elevation modulated transmission of α-syn 30 days after inoculation in the striatum. Immunohistochemical analysis showed that the immunoreactivity of phosphorylated α-syn in NeuN-positive cells of the striatum was markedly increased, whereas UA elevation in α-syn-inoculated mice significantly attenuated the immunoreactivity of phosphorylated α-syn in striatal neurons (Figure 3C). In addition, western blotting showed that UA elevation in α-syn-inoculated mice significantly decreased the expression of α-syn in the striatal region compared to only α-syn-inoculated mice (Figure 3D). ELISA analysis demonstrated that UA elevation in α-syn-inoculated mice significantly decreased the amount of phosphorylated α-syn in the striatum compared to α-syn-inoculated mice (Figure 3E). Furthermore, we assessed whether UA elevation led to microglia activation in the striatum of α-syn-inoculated mice. The expression of Iba-1 in the striatum was comparable among control mice, α-syn-inoculated mice, and UA-elevated mice (Figure 3F).

### UA elevation modulates α-syn propagation in the midbrain of α-syn-inoculated mice

**FIGURE 4:**
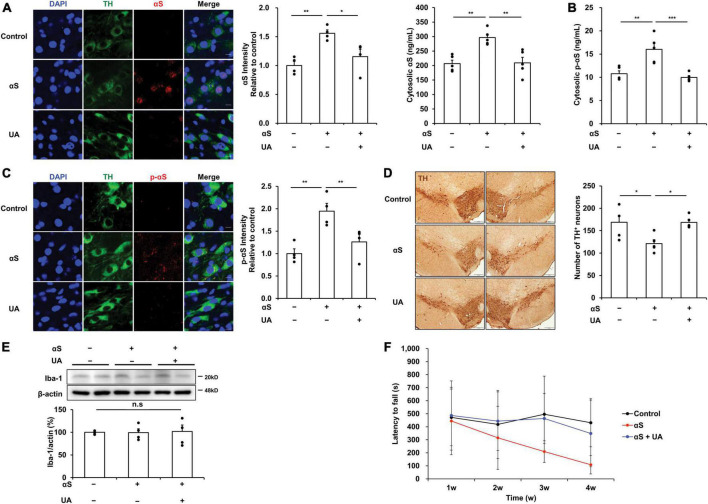
Uric acid (UA) elevation modulates α-syn propagation in the midbrain of α-syn-inoculated mice. **(A)** Immunostaining of αS co-localized with TH-positive neurons and quantification of fluorescence intensity ratio in the SNpc of midbrain. Immunoreactivity of αS was significantly decreased in UA-elevated mice compared to control and only αS-inoculated mice. Scale bar, 10 μm. **(B)** ELISA analysis showing that UA-treated mice had markedly decreased cytosolic p-αS in the midbrain compared to control and only αS-inoculated mice (*n* = 5 per group). **(C)** Immunostaining of phosphorylated αS co-localized with TH-positive neurons with quantification of fluorescence intensity ratio in the SNpc of midbrain. UA-elevated mice markedly attenuated the immunoreactivity of p-αS compared to control and only αS-inoculated mice with significant increase in TH-positive neurons. Scale bar, 5 μm. **(D)** Photomicrographs of SNpc of TH-immunostained sections. Graph shows the number of TH-positive cells in SNpc counted by stereology. Scale bar, 200 μm (*n* = 5 per group). **(E)** Western blotting showed that expression of Iba-1 in the midbrain was comparable among control, αS-inoculated, and UA-elevated mice (*n* = 4 per group). **(F)** UA treatment in α-syn-inoculated mice tended to improve motor performance on the rotarod test (*n* = 6, each group). Differences among conditions were evaluated by ANOVA with Bonferroni’s correction for multiple comparisons. All data are presented as mean ± SE. *P < 0.05, **P < 0.01, and ***P < 0.001.

Thirty days after inoculation of α-syn into the striatum, α-syn expression in the midbrain was significantly increased, as was the immunoreactivity of α-syn in dopaminergic neurons of the SN. However, UA elevation in α-syn-inoculated mice significantly attenuated the expression and immunoreactivity of α-syn in the midbrain compared to α-syn-inoculated mice (Figure 4A). Phosphorylated α-syn expression was examined using an ELISA analysis to evaluate whether exogenous α-syn induces pathogenic α-syn. Results indicated that α-syn inoculation markedly increased the levels of phosphorylated α-syn in the midbrain compared to control mice. However, UA elevation in α-syn-inoculated mice significantly attenuated phosphorylated α-syn levels compared to only α-syn-inoculated mice (Figure 4B). Phosphorylated α-syn was prominently immunostained with dopaminergic neurons of the SN in α-syn-inoculated mice, whereas the immunoreactivity of phosphorylated α-syn co-merged with dopaminergic neurons was markedly decreased in UA-elevated mice (Figure 4C). Consequently, α-syn inoculation led to a significant decrease in the number of TH-positive neurons in the SN compared to controls. However, UA elevation resulted in a significant increase in the number of TH-positive neurons (Figure 4D). Furthermore, the expression of Iba-1 in the midbrain was comparable among control, α-syn-inoculated, and UA-elevated mice (Figure 4E). Behavioral analysis showed that α-syn inoculation led to progressive patterns of the latency to fall on the rotarod test compared to the control group. However, UA treatment in α-syn-inoculated mice tended to improve motor coordination and balance on the rotarod test (Figure 4F).

### UA elevation modulates transmission of α-syn via blocking endocytic pathway in α-syn-inoculated mice

**FIGURE 5:**
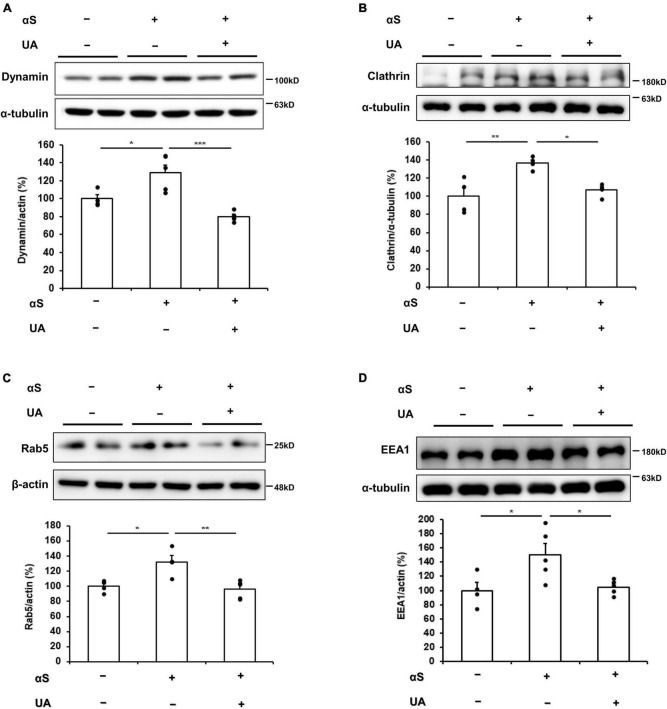
Uric acid (UA) elevation modulates transmission of α-syn via blocking endocytic pathway in α-syn–inoculated mice. **(A)** Western blot for dynamin in control (*n* = 4), αS (*n* = 5), αS/UA-treated (*n* = 5) neuronal cells, and quantification graph. **(B)** Western blot for clathrin and quantification graph (*n* = 4 per group). **(C)** Western blot for Rab5 and quantification graph (*n* = 4 for control, *n* = 5 for αS-inoculated and UA-elevated mice). **(D)** Western blot for EEA1 and quantification graph (*n* = 4 for control, *n* = 5 for αS-inoculated and UA-elevated mice). Differences among conditions were evaluated by ANOVA with Bonferroni’s correction for multiple comparisons. All data are presented as mean ± SE. *P < 0.05, **P < 0.01, and ***P < 0.001.

Next, we examined whether the elevation of UA levels modulated transmission of extracellular α-syn by blocking dynamin-mediated and clathrin-mediated endocytosis. In α-syn-inoculated mice, the expression of dynamin and clathrin in the midbrain markedly increased compared to control mice, whereas UA-elevated mice showed significantly decreased expression of dynamin and clathrin compared to only α-syn-inoculated mice (Figures 5A, B). In addition, UA elevation in α-syn-inoculated mice led to marked decrease in the expression of Rab5 localized to early endosome compared to only α-syn-inoculated mice (Figure 5C). Moreover, the expression of EEA1 significantly decreased in UA-elevated mice compared to only α-syn-inoculated mice (Figure 5D).

### α-syn modulation by UA elevation may not be associated with induction of autophagolysosome

**FIGURE 6:**
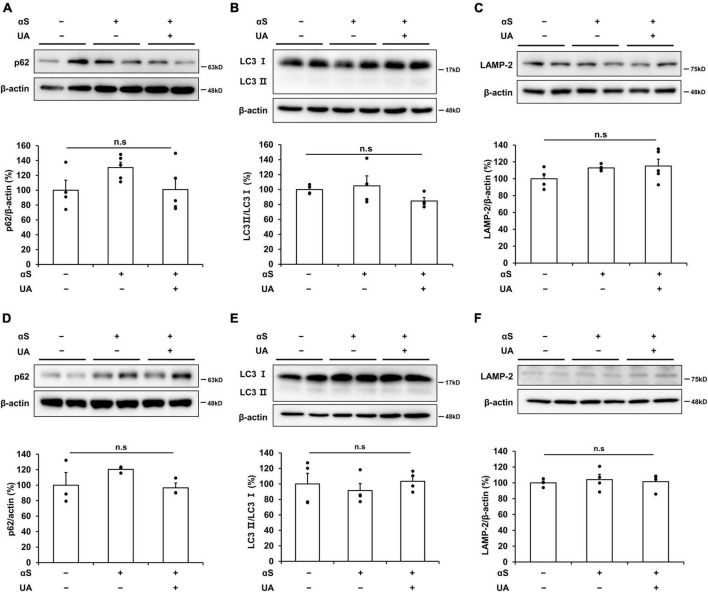
α-Syn modulation by UA elevation may not be associated with induction of autophagolysosome. **(A–C)** Western blots showing that expression of p62, LC3B, and LAMP-2 was comparable among the control (*n* = 4), αS (*n* = 5), and αS/UA-treated (*n* = 5) cells. **(D–F)** Western blots showing that expression of p62, LC3B, and LAMP-2 was comparable among control, αS-inoculated, and UA-elevated mice in the midbrain (*n* = 4 per group). Differences among conditions were evaluated by ANOVA with Bonferroni’s correction for multiple comparisons. All data are presented as mean ± SE.

Finally, we examined whether UA elevation induced the autophagic pathways to exclude the modulatory effect of UA on α-syn via autophagic clearance under the same experimental conditions. In a cellular model, α-syn incubation for 6 h did not induce any autophagolysosomal markers of p62, LC3B, or LAMP-2 compared to control neuronal cells. In addition, the expression of autophagy markers did not differ between α-syn-treated cells and UA-treated cells (Figures 6A–C). Similarly, UA elevation in α-syn-inoculated mice did not change the expression of p62, LC3B, or LAMP-2 in the midbrain compared to only α-syn-inoculated mice (Figures 6D–F). These data indicate that the effect of UA elevation on α-syn modulation may be associated with direct inhibition of α-syn propagation, but not due to autophagy-mediated α-syn clearance.

## Discussion

The present study investigated whether elevation of serum UA levels modulated the transmission of α-syn, resulting in a neuroprotective effect on dopaminergic neurons in Parkinsonian models. The major findings were: (1) UA inhibited cell-to-cell transmission of extracellular α-syn by modulating endocytosis; and (2) inhibition of α-syn propagation by the elevation of serum UA levels led to a pro-survival effect on nigral dopaminergic neurons in α-syn-inoculated mice. Our data suggest that UA may modulate the propagation of α-syn as an additive mechanism which controls the pathological conditions of PD.

There is ample evidence that neurons have an ability to internalize extracellular aggregates by endocytosis. Internalized α-syn aggregates can be transmitted from neuron-to-neuron via the extracellular milieu and can be propagated by a seeding mechanism (Guo and Lee, 2014; Lee et al., 2014; Tyson et al., 2017). Also, propagation of α-syn aggregates can induce neurotoxicity and neuronal loss in α-synucleinopathies, accompanied by impairment of motor and cognitive function (Conway et al., 1998; Forloni et al., 2000; Da Cunha et al., 2002; Volpicelli-Daley et al., 2011; Luk et al., 2012). Thus, the inhibition of α-syn propagation may be clinically relevant as a key pharmacological target for disease-modifying treatment for α-synucleinopathies. On this basis, our data suggested that the neuroprotective property of UA elevation in regulating the transmission of α-syn may be applicable to the development of future disease modifying strategies for patients with α-synucleinopathies.

The present study demonstrated that UA inhibits α-syn endocytosis and leads to the inhibition of α-syn transmission. In cellular models, UA inhibited the internalization of extracellular α-syn in an α-syn-enriched cellular environment and blocked neuron-to-neuron transmission of α-syn in donor-acceptor cell models. Previous report has demonstrated that the internalization of α-syn may be mediated by endocytosis and have shown that endocytosis inhibitors have an ability to decrease the internalization of α-syn both *in vitro* and *in vivo* (Hansen et al., 2011). Furthermore, in cultured cell lines, the overexpression of a dominant-negative dynamin efficiently reduces the extent of internalized α-syn aggregates (Lee et al., 2008, 2010) and suppression of dynamin GTPase decreases α-syn uptake by neuronal cells (Konno et al., 2012). In addition, clathrin-mediated endocytosis is another pathway of extracellular α-syn endocytosis. Studies have shown that α-syn interacts and co-localizes with components of the clathrin-coated pit (Erb and Moore, 2020; Schechter et al., 2020; Lee et al., 2021; Teixeira et al., 2021), suggesting that the endosomal pathway of α-syn may start with clathrin-mediated endocytosis. Our previous data also showed that suppression of clathrin-mediated endocytosis may lead to a reduction of internalized extracellular α-syn (Oh et al., 2016; Lee et al., 2021). In the present study, we found that α-syn fibrils were internalized into neuronal cells through dynamin-mediated and clathrin-mediated endocytosis, with up-regulated expression of dynamin and clathrin in α-syn-treated cells. However, UA-treated cells had significantly decreased expression of dynamin and clathrin as well as expression of Rab5. Small GTPases Rab5, a marker of early endosomes and regulator of endocytosis, is critical for the endocytosis of exogenous α-syn into neuronal cells (de Hoop et al., 1994; Stenmark et al., 1995; Neve et al., 1998; Sung et al., 2001). As a result, the expression of EEA1, a Rab5 effector protein, markedly decreased in UA-treated cells compared to cells treated with α-syn alone. Similarly, we found that UA treatment in α-syn-inoculated mice significantly decreased the expression of clathrin and dynamin in the midbrain, which subsequently decreased the expression of Rab5 and EEA1. Accordingly, the present study provides evidence that UA modulates extracellular α-syn propagation by inhibiting both dynamin-mediated and clathrin-mediated endocytosis.

We found that UA significantly decreased the levels of internalized cytosolic α-syn and attenuated α-syn-induced cell death in α-syn-enriched models. In cellular models, UA treatment decreased the expression of intracellular α-syn with an increase in the amount of extracellular α-syn. In addition, it has been reported that extracellular α-syn can act as seed that initiate the aggregation of endogenous α-syn, in both cellular and animal models (Mahul-Mellier et al., 2020; Awa et al., 2022). In our study, it was also observed that aggregated α-syn increased by extracellular α-syn was reduced by UA. This was possibly caused by inhibition of α-syn internalization, which led to a pro-survival effect in neuronal cells. In α-syn-inoculated mice, elevation of serum UA levels significantly decreased transmission of α-syn in the striatum as well as the midbrain region which is distant from the inoculation site. Consistent with the results of our *in vitro* study, the expression of several markers related to extracellular α-syn entry significantly decreased in UA-elevated mice inoculated with α-syn compared to mice that were inoculated with α-syn-alone. Specifically, 1 month after α-syn was inoculated into the dorsal striatum, UA elevation decreased the immunoreactivity and expression of the phosphorylated α-syn in the striatum and SN of midbrain. Thus, UA elevation in α-syn-inoculated mice resulted in increased survival of dopaminergic neuron in the SN. In terms of other mechanisms of UA on α-syn modulation, it is possible that reduction in the amount of α-syn could be secondary to modulation of autophagy or microglial activation because UA can enhance autophagy activity (Sheng et al., 2017) and inhibit microglia activation in PD model (Bao et al., 2018). Moreover, by blocking intracellular transmission, the subsequent increase in the burden of extracellular α-syn could lead to the induction of neuroinflammation (Gelders et al., 2018; Domingues et al., 2022). However, in the current study, we found that UA treatment did not lead to change in markers related with autophagolysosomal or microglial activity under the same experimental conditions. Accordingly, these data indicate that the beneficial effect of UA on α-syn modulation may be associated with direct modulation of α-syn propagation.

Although there are many studies on the beneficial effect of UA in neurodegenerative disease, serum uric acid concentrations above the saturation threshold promote the deposition monosodium urate crystals, leading to chronic inflammatory response such as goat, renal and cardiovascular diseases (Sautin and Johnson, 2008; So and Thorens, 2010; Vassalle et al., 2016). In the present study, when administered KOx 500 mg/kg and GMP 500 mg/kg for increasing UA, the serum uric acid level of UA-treated group was about 3.4 mg/dL, which was within the normal uric acid range of mice (serum urate concentration 0.1 to 760 μM (Watanabe et al., 2014). Given that UA can switch between having protective antioxidant capacity and having detrimental pro-oxidizing effects, depending on its concentration and the surrounding microenvironment, further laboratory and clinical studies are needed to uncover the optimum UA concentration for disease-modifying effect in PD.

## Conclusion

The present study demonstrated that UA has neuroprotective effects on dopaminergic neurons via the inhibition of extracellular α-syn transmission. Along with the pleiotropic effects of UA, repositioning use of UA focusing on α-syn propagation should be further investigated in patients with α-synucleinopathies.

## Data availability statement

The original contributions presented in this study are included in the article/Supplementary material, further inquiries can be directed to the corresponding author.

## Ethics statement

All procedures were performed in accordance with the Laboratory Animals Welfare Act, the Guide for the Care and Use of Laboratory Animals, and the Guidelines and Policies for Rodent Experiments provided by the Institutional Animal Care and Use Committee (IACUC) at the Yonsei University Health System. The study was conducted in accordance with the local legislation and institutional requirements.

## Author contributions

YS: conception and design, collection and/or assembly of data, manuscript writing, and final approval of manuscript. JEL, YSK, JWL, and HK: technical assistance. YJK and JS: conception and design, collection and/or assembly of data, and discussion. PL: supervision of study, data analysis and interpretation, financial support, and final approval of manuscript. All authors contributed to the article and approved the submitted version.
