# Supplementary figures and images for: Uric acid regulates α-synuclein transmission in Parkinsonian models
Source: Front Aging Neurosci. 2023 Aug 28;15:1117491. doi: 10.3389/fnagi.2023.1117491 (PMC10497982; doi:10.3389/fnagi.2023.1117491)

**Supplementary Figure 1.** Uncropped images of western blots displayed in Fig. 1F.

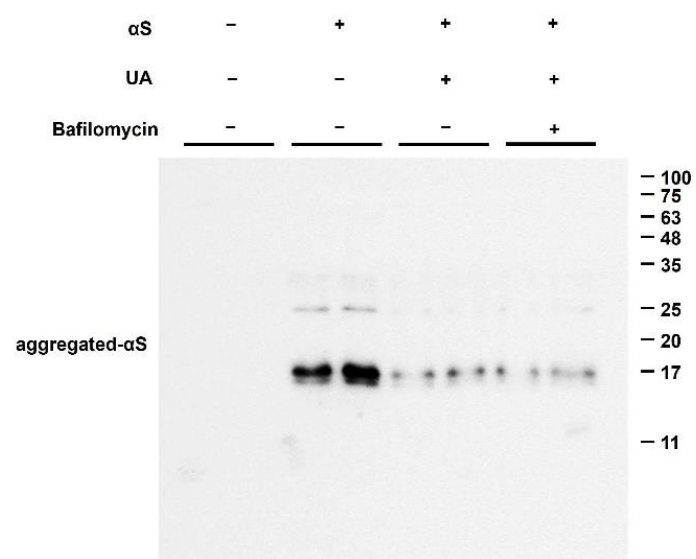

Supplement: Supplementary file 1 [file Image_1.PDF]
